# Financial Burden, Out-of-Pocket Health Spending, and Household Economic Well-Being in Heart Failure Patients in India: A Multicentre Cross-Sectional Survey

**DOI:** 10.5334/gh.1535

**Published:** 2026-03-12

**Authors:** Panniyammakal Jeemon, Reethu Salim, K. Safvan, Greeva Philip, Aditya Kapoor, Amir Rashid, Ajay Bahl, Animesh Mishra, Bhavesh Roy, Bishav Mohan, Dinesh Choudhary, Jabir Abdullakutty, Justin Paul, Jayesh Prajapati, Neelam Dahiya, Prakash C. Negi, Rishi Sethi, Satyanarayan Routray, Rajendiran Gopalan, P. Shyam Sunder Reddy, Veena Nanjappa, Meenakshi Sharma, Roopa Shivashankar, Sanjay Ganapathi, Sivadasanpillai Harikrishnan

**Affiliations:** 1 Sree Chitra Tirunal Institute for Medical Sciences and Technology, Trivandrum, Kerala, India; 2 ICMR-Centre for Advanced Research and Excellence in Heart Failure (CARE-HF), Thiruvananthapuram, Kerala, India; 3 Sanjay Gandhi Post graduate Institute of medical sciences, Lucknow, India; 4 Sher-i-Kashmir Institute of Medical Sciences, Srinagar, Jammu and Kashmir, India; 5 Post Graduate Institute of Medical Education and Research, Chandigarh, India; 6 North Eastern Indira Gandhi Regional Institute of Health and Medical Sciences, Shillong, India; 7 Zydus Hospital, Ahmedabad, India; 8 Dayanand Medical College Hospital, Ludhiana, India; 9 Sardar Patel Medical College, Bikaner, India; 10 Lisie Hospital, Ernakulam, India; 11 Madras Medical College, Chennai, India; 12 U. N. Mehta Institute of Cardiology & Research Centre, Ahmedabad, India; 13 Indira Gandhi Medical College Hospital, Shimla, Himachal Pradesh, India; 14 King George’s Medical University, Lucknow, India; 15 Medical College, Cuttack, India; 16 Institute of Medical Sciences and Research, Coimbatore, India; 17 Hospitals, Kondapur, Hyderabad, India; 18 Sri Jayadeva Institute of Cardiovascular Science and Research, Mysore, Karnataka, India; 19 Division of Non-Communicable Diseases, Indian Council of Medical Research, New Delhi, India

**Keywords:** financial burden, catastrophic health spending, distress financing, social insurance, household economic wellbeing

## Abstract

**Background:**

Heart failure (HF) is a complex clinical condition requiring resource-intensive management and substantial health expenditure. The adverse economic impact of medical care on patients or financial burden is increasingly recognised as a significant non-clinical entity affecting HF management in low- and middle-income countries (LMIC). We explored the factors associated with Financial Burden (FB) in HF patients in India.

**Methods:**

We recruited HF patients from 21 hospitals across India, selected to reflect regional diversity and varying stages of epidemiological transition. Trained personnel collected clinical and economic data using a validated and structured questionnaire. Expenditures were recorded in Indian rupees (INR) and converted to international dollars (INT$).

**Results:**

We recruited 1,859 participants. Nearly one-third of participants (30.2%) were women. The mean age was 55.9 (11.3) years, and the mean duration of formal education was 11.3 (3.8) years. Health insurance coverage was reported in one-third (32.2%) of the study population. The average annual out-of-pocket (OOP) expenditure was INR 1,06,566 (INT$ 4,709.10), constituting 92.6% (95% CI: 92.5–92.7) of the total health expenditure. Compared to the previous year, a decline in monthly income was reported by 32.3% of individuals and 36.2% of households. Catastrophic health spending (CHS) and distress financing (DF) were observed in 37.7% (35.5–39.9) and 17.7% (15.9–19.4) of the households, respectively. However, CHS and DF were lower [30.8% (26.2–35.4) and 13.6% (10.2–17.0), respectively] among those with health insurance compared to the uninsured [40.3% (37.6–43.0) and 18.9% (16.7–21.1), respectively].

**Conclusion:**

Seven out of 10 HF patients in India lack financial health protection. OOP expenditures, accounting for over 90% of total health spending, contribute significantly to economic distress in HF patients. Financial burden, affecting more than one-third of HF patients, carries profound implications for individual well-being. Addressing this financial burden, including CHS and DF, is essential for improving clinical outcomes and ensuring health equity.

## Introduction

Heart failure (HF) affects an estimated 26 million people worldwide, with its burden increasing steadily in low- and middle-income countries (LMICs) (1). In India alone, HF accounts for 1.8 million hospitalisations annually and places a considerable burden on the health system due to prolonged inpatient care and the need for long-term management, straining already limited healthcare resources. Further, due to its debilitating nature, HF is also known for its massive financial burden on patients, caregivers, and society (1).

Despite improved insurance coverage through schemes like Ayushman Bharat, the financial impact of cardiovascular diseases (CVDs), particularly HF, remains substantial for economically vulnerable households in India (2). The Ayushman Bharat scheme provides an annual health insurance coverage of ₹5 lakhs (INT$22K) per family for secondary and tertiary care hospitalisation, focusing mainly on economically disadvantaged populations (3). By extending coverage to a large section of the population, Ayushman Bharat promotes universal health coverage and lowers out-of-pocket (OOP) expenditure. However, gaps remain in awareness, enrolment, and access, particularly for chronic conditions like HF. In a study from Manipal, the average cost per HF hospitalisation was approximately ₹1.34 lakhs (INT$5.9K), of which 62% was OOP expenses (4). Similarly, in a study from Punjab, CVD conditions incurred the highest inpatient and outpatient costs among non-communicable diseases.

HF is associated with high costs due to the need for long-term medications, repeated hospitalisations, and specialised care, especially during acute episodes. These costs often result in financial burden, distress, and toxicity, particularly in the absence of comprehensive insurance. Financial burden refers to the comprehensive economic strain, encompassing both direct medical costs (hospitalisations, medications, devices) and indirect costs (lost productivity, caregiver time), which affects patients, families, and health systems (5). The current understanding of financial burden in chronic disabling conditions, such as HF, in the Indian context remains limited. Most existing data are from small, region-specific studies. There is a lack of national-level evidence on OOP spending, catastrophic health spending (CHS), distress financing (DF), and their contribution to financial burden among HF patients. This study aimed to generate evidence on the financial burden faced by HF patients in India, to inform health policy strategies that strengthen financial protection, advance universal health coverage, and alleviate the economic burden on vulnerable populations.

## Methodology

The detailed methodology of our study is published elsewhere (6). In brief, consecutive HF patients were enrolled from 21 tertiary care hospitals in India from September 2019 to December 2022. Hospital sites were selected to represent diverse regions (states) based on the epidemiological transition levels (ETL), defined as the ratio of all-age disability-adjusted life years (DALYs) attributable to communicable, maternal, neonatal, and nutritional diseases (CMNNDs) to those resulting from non-communicable diseases and injuries. States were categorised as having low (0.56–0.75), lower-middle (0.41–0.55), higher-middle (0.31–0.40), or high (<0.31) ETLs. This study combined low and lower-middle ETL states (0.41–0.75) into a single group, while higher-middle and high ETLs were retained as separate categories. Six to eight hospitals were selected from each of the three ETL-based regions.

We included HF patients whose most recent hospitalisation occurred 6–15 months before data collection. Limiting data collection to the period after the acute admission date enabled us to collect inpatient, procedural, and outpatient costs from all study participants. The recruitment target was 1,800 participants, 600 from each of the three ETL-based regions, which ensured 80% power to detect an 8% absolute difference (50% and 58%) in the primary outcome, defined as OOP expenditure as a proportion of total health expenditure, between any two groups.

The Sree Chitra Tirunal Institute for Medical Sciences and Technology, Trivandrum (SCTIMST), coordinated the study across the selected centres. The institutional ethics committee (IEC) of SCTIMST (SCT/IEC/1313.6/DECEMBER-2018) approved the study protocol. Further, we obtained approval from the IECs of individual participating centres. Written informed consent was obtained from all participants before data collection.

### Study measurements

We adapted an existing structured interview schedule previously employed in an economic assessment study conducted in India (7). It covered access to health insurance, treatment costs, productivity, household demographics, overall expenditures, household assets, and financing mechanisms. The modified study tool underwent a rigorous validation process. We sought feedback from both subject matter experts and patients to establish face validity. For translational validity, the questionnaire was independently reviewed in English and regional languages by experts. We then piloted the questionnaire with 10 patients or their caregivers, carefully observing their responses to each item to assess clarity and relevance. The final document was translated into 10 additional Indian languages, based on the planned recruitment locations across different states. Another structured questionnaire obtained detailed information on clinical parameters and functional status. The study tools were developed with input from a multidisciplinary team of experts in cardiology, epidemiology, health economics, and HF patients (6).

During follow-up visits, we collected data from consecutive patients on history, treatment, clinical parameters, household healthcare expenditures, OOP expenses, financing mechanisms, and the potential impoverishing effects of healthcare spending. Trained field staff conducted face-to-face interviews in the participants’ regional languages. All expenditure data were recorded in Indian rupees (INR). Medication adherence was assessed using two specific questions: whether participants had obtained their prescribed medications and, if so, the reasons for missing doses.

### Operational definitions

We calculated in-hospital admission costs as the sum of self-reported spending on various components, including hospital admission charges, emergency room fees, treatment-related costs (such as room rent, surgical or intervention procedures, and medications), diagnostic investigations (blood tests, imaging, electrocardiogram, echocardiography), food, travel to and from the hospital, and attendant-related costs (food, accommodation, and travel). Post-hospitalisation costs include expenditures on physician and caregiver fees, further investigations (blood tests, ECG, echocardiography, and other tests), medications, food, and transportation. Information on reimbursements received from third-party payers was also obtained.

Annual household expenditure was estimated by aggregating self-reported yearly spending on rent, food, transportation, education, household goods, clothing, cooking fuel, healthcare, vehicle maintenance, property upkeep, and other miscellaneous expenses. The data were collected for the previous month and annualised for items such as rent, food, transportation, education, and outpatient healthcare (including medication purchases). For all other categories, the reference period was one year.

Total OOP expenditure on health comprised all direct and selected indirect health-related costs (6). A household’s health expenditure was classified as catastrophic if annual OOP spending on health equalled or exceeded 40% of its capacity to pay (8). Capacity to pay was defined as the total household expenditure (THHE) minus subsistence expenditure (SE), representing non-subsistence spending. Participants were identified as having undergone ‘distress financing’ if they reported borrowing money from friends, relatives, or financial institutions, or selling/mortgaging assets to meet healthcare expenses (7). DF also included informal loans without interest.

All expenditures were recorded in Indian rupees (INR) and converted into 2021 international dollars using purchasing power parity, with an exchange rate of 22.63 for 1 USD (9). Unless otherwise indicated, all costs reported herein as ‘INT$’ represent international dollars.

### Data quality checks

The data underwent a systematic quality assurance process to ensure accuracy, completeness, and internal consistency. An experienced data manager assessed the dataset for missing values, duplicate entries, incorrect formats, and logical inconsistencies. Any discrepancies or inaccuracies were flagged and sent back to the respective centres for correction. Upon receiving the revised data, the data manager reviewed and verified the corrections using supporting documentation. The final datasets were prepared in consultation with the study statistician. All data management procedures were completed before the database lock. Following approval from all investigators, the database was formally locked, and the final dataset was extracted for statistical analysis.

### Statistical analyses

Continuous variables were described using means and standard deviations (if normally distributed), while categorical variables were expressed as frequencies and percentages (%). For skewed continuous variables, both the mean with standard deviation and the median with interquartile range were reported.

Cluster-adjusted generalised estimating equation (GEE) and mixed-effects logistic regression analyses were performed to identify factors associated with CHS and DF, with clustering defined at the hospital level. Both models were adjusted for relevant clinical and socioeconomic covariates, including age, sex, New York Heart Association (NYHA) functional class, and epidemiological transition level (ETL). Adjusted odds ratios (aORs) with 95% confidence intervals (CIs) were presented. Statistical significance was defined as a two-sided p-value below 0.05 unless stated otherwise.

## Results

We recruited 1,859 participants across three epidemiological transition level (ETL) categories: higher ETL (n = 666), higher-middle ETL (n = 600), and low and lower-middle ETL (n = 593). The mean age of the participants was 55.9 years (SD: 11.3), and 30.2% of the study population were women. The average duration of formal education was 11.3 years (SD: 3.8). Approximately 48.6% of the participants resided in rural areas (Table 1).

**Table 1:** Socio-demographic details and clinical history of study participants.


VARIABLES	INSURANCE (n = 600)	NO INSURANCE (n = 1259)	TOTAL (n = 1859)

Age in years, mean (SD)	55.64 (11.41)	56.09 (11.18)	55.95 (11.26)

Male, n (%)	416 (69.33)	882 (70.06)	1298 (69.82)

Female, n (%)	184 (30.67)	377 (29.94)	561 (30.18)

Currently married, n (%)	516 (86.00)	1121 (89.04)	1637 (88.06)

Years of education, mean (SD)	11.51 (3.83)	11.23 (3.79)	11.32 (3.81)

Employed, n (%)	395 (65.83)	823 (65.37)	1218 (65.52)

Unemployed (including students and homemakers), n (%)	205 (34.17)	436 (34.63)	641 (34.48)

Duration of hospital stay, median (Q1, Q3)	5 (4, 8)	5 (4, 8)	5.00 (4.00, 8.00)

Rural, n (%)	296 (49.33)	607 (48.21)	903 (48.6)

Diabetes, n (%)	220 (36.7)	468 (37.2)	688 (37.0)

Hypertension, n (%)	259 (43.2)	519 (41.2)	778 (41.9)


SD: Standard deviation, Q1: lower quartile part, Q3: upper quartile part.

Ischemic heart disease was the predominant aetiology, observed in 74.3% of participants. Dilated cardiomyopathy and rheumatic heart disease were present in 17.1% and 3.4% of participants, respectively. Comorbid diabetes mellitus was reported by 37.0% of participants, while 41.9% reported a history of hypertension. Tobacco use was prevalent in 16.9% of the study population (Table 1). Nearly two-fifths (39.5%) of participants obtained their medications from government-run pharmacies.

The average self-reported monthly individual income was INR 7,866.2 (INT$347.6). Similarly, the average household monthly income was INR 27,805.0 (INT$1,228.7). A reduction in monthly income following HF diagnosis was reported by 32.3% (30.2, 34.4) of individuals and 36.2% (34.0, 38.4) of households (Table 2).

**Table 2:** Expenditure pattern and financing measures in the study population.


VARIABLES	INSURANCE (n = 600)	NO INSURANCE (n = 1259)	TOTAL (n = 1859)

Total hospitalisation expenditure, median (Q1, Q3)	INR: 51600 (27100, 125750) INT$: 2283.2 (1199.1, 5564.2)	INR: 48000 (20270, 143000) INT$: 2123.9 (896.9, 6327.4)	INR: 49600 (23500, 135000) INT$: 2194.7 (1039.8, 5973.5)

Total hospitalisation expenditure, mean (SD)	INR: 103637 (129828) INT$: 4579.6 (5744.6)	INR: 126254 (191435) INT$: 5579.1 (8470.6)	INR: 118954 (174238) INT$: 5256.5 (7709.6)

Total expenditure on food, median (Q1, Q3)	INR: 5000 (3000, 8000) INT$: 221.2 (132.7, 353.9)	INR: 5000 (2500, 8000) INT$: 221.2 (110.6, 353.9)	INR: 5000 (2500, 8000) INT$: 221.2 (110.6, 353.9)

Total expenditure on food, mean (SD)	INR: 6412.1 (6804) INT$: 283.34 (301.1)	INR: 29734 (845373) INT$: 1313.92 (37405.9)	INR: 22206 (695707) INT$: 981.30 (30783.5)

Total out-of-pocket expenditure, median (Q1, Q3)	INR: 39842 (20525, 82000) INT$: 1762.9 (908.2, 3628.3)	INR: 45200 (20000, 140500) INT$: 2000 (884.9, 6216.8)	INR: 43000 (20100, 115000) INT$: 1902.7 (889.4, 5088.5)

Total out-of-pocket expenditure, mean (SD)	INR: 70869 (97919) INT$: 3131.6 (4332.7)	INR: 123580 (190211) INT$: 5460.9 (8416.4)	INR: 106567 (167915) INT$: 4709.1 (7429.9)

**Distress financing measures**			

Own savings, n (%)	423 (70.5)	836 (66.4)	1259 (67.7)

Family member paid, n (%)	317 (52.8)	694 (55.1)	1011 (54.4)

Employer paid, n (%)	7 (1.2)	16 (1.3)	23 (1.2)

Borrowed from friends, family, and employer, n (%)	72 (12)	199 (15.8)	271 (14.6)

Borrowed from banks, moneylenders, n (%)	33 (5.5)	109 (8.7)	142 (7.6)

Sold house, land, or other assets, n (%)	8 (1.3)	62 (4.9)	70 (3.8)

**Household expenditure**			

Decrease in individual monthly income, n (%)	204 (34.00)	396 (31.45)	600 (32.3)

Baseline monthly individual income before heart failure, median (Q1, Q3)	INR: 4000 (500, 20000) INT$: 176.9 (22.6, 884.9)	INR: 5000 (350, 20000) INT$: 221.2 (15.5, 884.9)	INR: 5000 (400, 20000) INT$: 221.2 (17.7, 884.9)

Baseline monthly individual income before heart failure, mean (SD)	INR: 13229.2 (21039.4) INT$: 584.6	INR: 12293.5 (16307.3) INT$: 543.2	INR: 12595.5 (17970.8) INT$: 556.6

Current monthly individual income in INR, median (Q1, Q3)	INR: 2000 (200, 10000) INT$: 88.5 (8.9, 442.5)	INR: 1800 (100, 10000) INT$: 79.6 (4.4, 442.5)	INR: 1950 (150, 10000) INT$: 86.3 (6.6, 442.5)

Current monthly individual income, mean (SD)	INR: 8016.8 (14474.3) INT$: 354.3 (640.5)	INR: 7794.6 (12189.3) INT$: 344.4 (539.3)	INR: 7866.2 (12966.5) INT$: 347.6 (573.7)

Decrease in individual monthly household income, n (%)	195 (32.50)	478 (37.97)	673 (36.2)

Baseline monthly household income before heart failure in INR, median (Q1, Q3)	INR: 30000 (15000, 45000) INT$: 1327.4 (663.7, 1991.2)	INR: 26000 (15000, 45000) INT$: 1150.4 (663.7, 1991.2)	INR: 28000 (15000, 45000) INT$: 1238.9 (663.7, 1991.2)

Baseline monthly household income before heart failure, mean (SD)	INR: 33295.4 (34537.6) INT$: 1471.3 (1528.2)	INR: 31932.6 (26875.9) INT$: 1411.1 (1189.2)	INR: 32372.4 (29563.9) INT$: 1430.5 (1306.9)

Current monthly household income in INR, median (Q1, Q3)	INR: 25000 (10000, 40000) INT$: 1106.2 (442.5, 1769.9)	INR: 20000 (10000, 40000) INT$: 884.9 (442.5, 1769.9)	INR: 25000 (10000, 40000) INT$: 1106.2 (442.5, 1769.9)

Current monthly household income	INR: 30051.0 (34858.6) INT$: 1327.9 (1542.4)	INR: 26735.6 (26062.9) INT$: 1181.4 (1153.2)	INR: 27805.7 (29224.4) INT$: 1228.7 (1293.1)


SD: Standard deviation, Q1: lower quartile part, Q3: upper quartile part, INR: Indian Rupees, INT$: International Dollar.

The median length of hospital stay for the last hospitalisation was 5.0 days (IQR 3.0–7.0). The average hospitalisation cost was INR 1,18,954.21 (INT$5,256.5). It was not different in men (INR 1,18,042.5) and women (INR 1,21,063.7).

The average out-of-pocket health spending (OOPS) was INR 1,06,566.9 (INT$4,709). OOP health expenditure accounted for 92.6% (95% CI: 92.5–92.8) of the total health spending. The reported sources of payment for OOPS included personal savings in 67.7% of participants (95% CI: 65.6–69.9), financial support from the family in 54.4% (95% CI: 52.1–56.7), and borrowing from family and friends in 14.6% (95% CI: 13.0–16.2).

**Figure 1:**
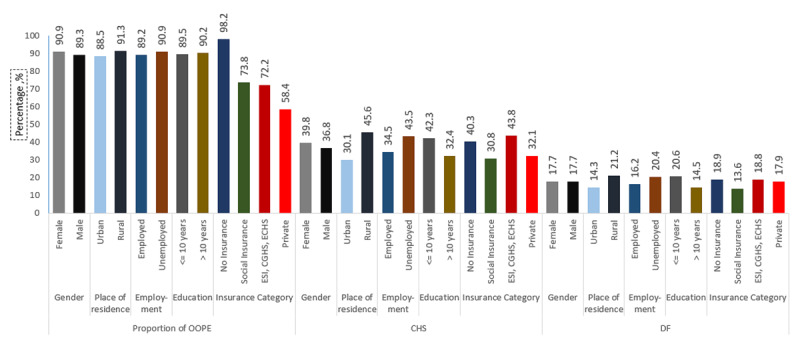
Economic Impact of heart failure in India. OOPE: Out of pocket Expenditure, CHS: Catastrophic health spending, DF: Distress financing.

CHS was reported by 700 participants (37.7%; 95% CI: 35.5–39.9). The prevalence of CHS was higher among females (39.8%), individuals living in rural areas (45.6%), unemployed individuals (43.5%), and participants with less than secondary education (42.4%) (Table 3 and Figure 1). DF was reported by 17.7% (16.0–19.4) of participants. Individuals living in rural areas (21.3%) and unemployed individuals (20.4%) reported relatively higher DF (Figure 1). Similarly, individuals with lower educational attainment (less than secondary school) experienced a relatively higher prevalence of DF (20.6%) (Figure 1).

**Table 3:** Characteristics of participants with and without distress financing and catastrophic health spending.


VARIABLES	DF, n = 329	NO DF, n = 1530	CHS, n = 700	NO CHS, n = 1159	BOTH CHS AND DF, n = 120	WITHOUT BOTH CHS AND DF, n = 1739

Male, n (%)	230 (17.7)	1068 (82.3)	477 (36.7)	821 (63.3)	85 (6.5)	1213 (93.5)

Female, n (%)	99 (17.6)	462 (82.4)	223 (39.8)	338 (60.2)	35 (6.2)	526 (93.8)

Urban, n (%)	137 (14.3)	819 (85.7)	288 (30.1)	668 (69.9)	34 (3.6)	922 (96.4)

Rural, n (%)	192 (21.3)	711 (78.7)	412 (45.6)	491 (54.4)	86 (9.5)	817 (90.5)

Employed, n (%)	198 (16.3)	1020 (83.7)	421 (34.6)	797 (65.4)	69 (5.7)	1149 (94.3)

Unemployed, n (%)	131 (20.4)	510 (79.6)	279 (43.5)	362 (56.5)	51 (8.0)	590 (92.0)

Education < 10 years, n (%)	201 (20.6)	774 (79.4)	413 (42.4)	562 (57.6)	83 (8.5)	892 (91.5)

Education > 10 years, n (%)	128 (14.5)	756 (85.5)	287 (32.5)	597 (67.5)	37 (4.2)	847 (95.8)

More than one hospitalisation, n (%)	66 (22.2)	231 (77.8)	139 (46.8)	158 (53.2)	30 (10.1)	267 (89.9)

Only one hospitalisation, n (%)	258 (17.8)	1194 (82.2)	554 (38.2)	898 (61.8)	90 (6.2)	1362 (93.8)

High socioeconomic status, n (%)	1 (3)	32 (97)	4 (12.1)	29 (87.9)	0 (0)	33 (100)

Middle socio-economic status, n (%)	113 (15.4)	619 (84.6)	228 (31.1)	504 (68.9)	35 (4.8)	697 (95.2)

Lower socioeconomic status, n (%)	215 (19.7)	879 (80.3)	468 (42.8)	626 (57.2)	85 (7.8)	1009 (92.2)

Higher ETL, n (%)	110 (16.5)	556 (53.5)	310 (46.5)	356 (53.4)	55 (8.3)	611 (91.7)

Higher Middle ETL, n (%)	87 (15.1)	487 (84.9)	186 (32.4)	358 (67.6)	25 (4.4)	549 (95.6)

Low and lower middle ETL, n (%)	132 (21.3)	487 (78.7)	204 (32.9)	415 (67)	40 (6.5)	579 (93.5)


*ETL: epidemiological transition level, CHS: catastrophic health spending, DF: distress financing.

Among participants without health insurance, OOPS accounted for 97.9% (95% CI: 97.8–98.0) of total health expenditure (Table 4). In comparison, the proportion of OOPS was lower among those covered by social insurance (73.8%) and private insurance (58.5%). The prevalence of CHS was 40.3% (37.6–43.0) among uninsured participants, whereas it was lower at 30.8% (26.2–35.4) among participants with social insurance (Table 4).

**Table 4:** Total expenditure and out-of-pocket expenditure pattern in the study population.


VARIABLES	NO INSURANCE (n = 1259)	SOCIAL INSURANCE (PMJAY & SHI) (n = 390)	ESI, CGHS, ECHS (n = 48)	PRIVATE INSURANCE (n = 162)

Total IP expenses, mean, INR(USD)	101155.48(4469.97)	63699.57(2814.83)	110845.10(4898.15)	110427.30(4879.69)

Total OP expenses, mean, INR(USD)	20809.32(919.55)	21420.78(946.57)	25919.09(1145.34)	16716.09(738.67)

Total expenditure, mean, INR(USD)	126253.94(5579.05)	86320.59(3814.43)	140900.23(6226.26)	134283.52(5933.87)

Total OOPE, mean, INR(USD)	123579.66(5460.88)	63740.49(2816.64)	102983.54(4550.75)	78513.30(3469.43)

Proportion of OOPE	97.88	73.84	73.09	58.47

CHS, n (%)	507 (40.3)	120 (30.8)	21 (43.8)	52 (32.1)

Distress financing, n (%)	238 (18.9)	53 (13.6)	9 (18.8)	29 (17.9)


*IP: in-patient, OP: out-patient, OOPE: out-of-pocket expenditure, CHS: catastrophic health spending, PMJAY: Pradhan Mantri Jan Arogya Yojana, SHI: state health insurance (specific for each state), ESI: Employee state insurance, CGHS: Central Government Health Scheme, ECHS: Ex-Servicemen Contributory Health Scheme.

**Figure 2:**
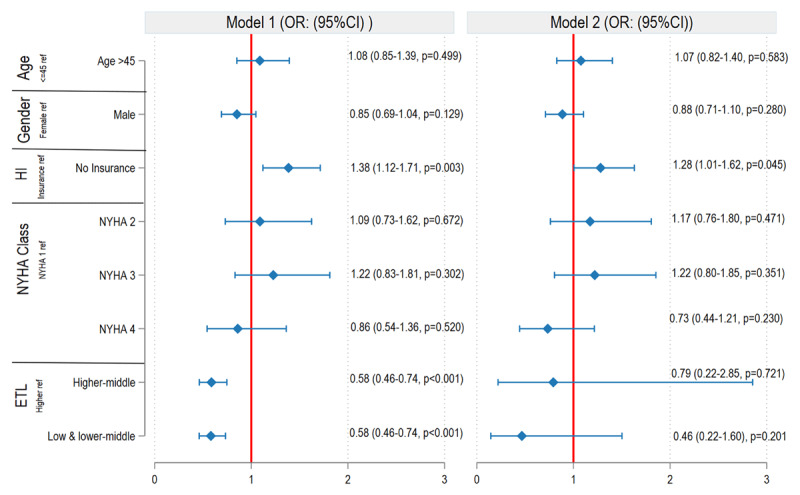
Multivariable regression analysis of catastrophic health spending. Model 1: Cluster-adjusted generalised estimating equation (Cluster GEE) adjusted for age, gender, health insurance status, NYHA classification, and epidemiological transition level (ETL). Model 2: Mixed-effects logistic regression adjusted for age, gender, health insurance status, NYHA classification, and epidemiological transition level (ETL). HI: Health insurance, NYHA: New York Heart Association Functional Classification.

**Figure 3:**
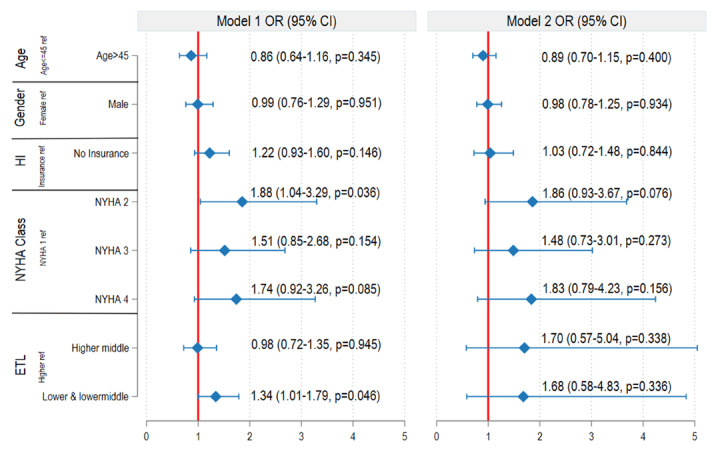
Multivariable logistic regression analysis of distress financing. Model 1: Cluster-adjusted generalised estimating equation (Cluster GEE) adjusted for age, gender, health insurance status, NYHA classification, and epidemiological transition level (ETL). Model 2: Mixed-effects logistic regression adjusted for age, gender, health insurance status, NYHA classification, and epidemiological transition level (ETL). HI: Health insurance, NYHA: New York Heart Association Functional Classification.

In both the mixed-effects logistic regression and the cluster-adjusted GEE analyses, the absence of insurance was associated with higher CHS than with insurance. The mixed-effects model showed an aOR of 1.28 (95% CI: 1.01–1.62; p = 0.045), whereas the GEE model showed a relatively larger effect size (aOR: 1.38; 95% CI: 1.12–1.71; p = 0.003).CHS was substantially higher in the high ETL group than in the low, middle, and higher-middle ETL groups (Figure 2). In the cluster-adjusted GEE model for DF (Figure 3), the absence of insurance was associated with an aOR of 1.22 (95% CI: 0.93–1.60; p = 0.146), whereas the mixed-effects logistic regression model yielded an aOR of 1.03 (95% CI: 0.72–1.48; p = 0.844).

## Discussion

We conducted this multicentre study across 21 institutions selected from different regions of India to assess the economic impact of HF at the individual and household levels. The study population, with a mean age of 55 years, was at least 10–15 years younger than the Western (10,11) or Chinese HF registries (12), highlighting the impact of the disease on people in their most productive years. OOP spending accounted for over 90% of total health expenditure, and 38% of participants experienced CHS, and 18% reported DF. Lower socio-economic status, rural residence, lack of insurance, and multi-morbidity were key predictors of financial hardship. Our findings highlight the significant economic and societal burden of HF in India.

One out of three individuals and households reported a decline in annual income following an HF diagnosis in our study. Similar income reductions have been observed in economic assessments of other chronic conditions (13). This decline may result from the patient’s inability to continue working due to illness, or from a family member being forced to cut short working hours or stop working to care of the patient. Evidence from multiple countries suggests that the rising burden of chronic diseases, such as CVD, diabetes, and mental health disorders, negatively affects labour force participation (13). Reduced income, in turn, can limit access to treatment, worsen clinical outcomes, and diminish overall quality of life.

In our study, two of five households experienced CHS, and one in six reported DF, indicating a substantial financial burden due to HF. Such expenditures often force families to reduce spending on essential needs, particularly among those with lower incomes, where even modest healthcare costs can be unaffordable. Heart failure, as a chronic and progressively worsening condition, requires frequent hospitalisations and continuous medical care, contributing to high OOP expenses (12). This aligns with prior evidence showing that the risk of CHS is closely tied to the share of OOP spending (4). Multiple factors contribute to financial burden among individuals with HF, with a decline in household income being a key driver. As noted earlier, nearly one-third of our participants experienced reduced income, which may have exacerbated their financial vulnerability.

Despite relatively low coverage, health insurance emerged as an important financial protection mechanism for people living with HF. Only about one-third of participants reported any form of insurance. Insurance coverage, particularly through schemes such as PMJAY and State Health Insurance, was associated with lower OOP expenditure and reduced CHS and DF. However, most existing insurance schemes are largely limited to inpatient care, leaving outpatient visits and long-term medication costs uncovered (14). This structural gap contributes substantially to financial burden, reflected in poor medication adherence and persistent economic strain after hospital discharge. Given that HF is a progressive condition requiring lifelong treatment, regular follow-up, and repeat hospitalisation events (15), the financial burden continues even among insured individuals, while those without insurance face a disproportionate risk of financial distress.

Expanding national insurance schemes to cover outpatient care and essential medications could substantially reduce the financial burden. Strengthening publicly funded insurance, improving access to free medicines through primary care, and including all guideline-directed HF therapies in the Essential Medicines List would further enhance affordability, reduce OOP expenditure, and promote equity in long-term HF care (16,17).

The primary drivers of CHS include rising healthcare costs and inadequate insurance coverage, both of which increase OOP payments (20,21). OOP health expenditures are a well-documented cause of financial impoverishment, pushing households across all income levels below the poverty line (18). Low socioeconomic status is a major predictor of CHS; individuals from these groups often have limited access to timely care and face worse health outcomes, including premature mortality (19,20). Our findings reinforce existing literature linking low income and rural residence to higher CHS risk (11). Notably, participants from rural areas reported substantially greater financial hardship than their urban counterparts.

## Strengths and limitations

We embedded this study within the National HF Registry (NHFR), one of the largest HF registries in India, with robust representation from all geographic regions (21). Using a standardised methodology for expenditure data collection across the study population enhanced internal validity. Conducting data collection during the same period at all sites facilitated meaningful comparisons and reliable pooling of data. The large-scale economic impact assessment, coupled with the inclusion of HF patients from across the country, strengthened the external validity of our findings. Furthermore, including both public and private healthcare centres in the sample provided a balanced perspective and further enhanced the generalisability of the results.

Our study is subject to several important limitations. Our findings may be influenced by the non-representativeness of the sample, as we did not account for individuals who forewent medical treatment entirely due to financial constraints or those who could not attend hospital follow-up due to the severity of their illness. Survival bias is another limitation, as the analysis includes only participants who returned for follow-up. Furthermore, estimates of OOP and CHS relied on self-reported expenditure data, which is susceptible to recall bias. Methodologically, the lack of adjustment for clustering (e.g., at the hospital or regional level) may have affected the precision of our estimates. Finally, as expenditure was assessed only immediately after a hospital admission for HF, this represents a limited assessment of financial burden and may not capture the cumulative, long-term economic burden experienced by patients and their families.

## Conclusion

In India, seven out of 10 patients with HF lack any form of financial health protection. With OOP expenses comprising over 90% of total health spending, the economic burden on HF patients is substantial, leading to financial burden in more than one-third of cases. This economic strain has profound implications for individual well-being and adversely affects treatment adherence, clinical outcomes, and population-level health equity. Addressing CHS and associated DF in chronic, debilitating conditions such as HF constitutes a critical public health priority, necessitating policy interventions that enhance financial risk protection and ensure equitable access to affordable, guideline-directed care. Future research should explore sustainable financing models and evaluate the impact of expanded insurance coverage and community-based care strategies on reducing financial burden in HF patients.
